# Prevalence of nonsuppressed viral load and associated factors among HIV‐positive adults receiving antiretroviral therapy in Eswatini, Lesotho, Malawi, Zambia and Zimbabwe (2015 to 2017): results from population‐based nationally representative surveys

**DOI:** 10.1002/jia2.25631

**Published:** 2020-11-22

**Authors:** Andreas D Haas, Elizabeth Radin, Avi J Hakim, Andreas Jahn, Neena M Philip, Sasi Jonnalagadda, Suzue Saito, Andrea Low, Hetal Patel, Amee M Schwitters, John H Rogers, Koen Frederix, Evelyn Kim, George Bello, Daniel B Williams, Bharat Parekh, Karampreet Sachathep, Danielle T Barradas, Thokozani Kalua, Sehin Birhanu, Godfrey Musuka, Owen Mugurungi, Beth A Tippett Barr, Katrina Sleeman, Lloyd B Mulenga, Kyaw Thin, Trong T Ao, Kristin Brown, Andrew C Voetsch, Jessica E Justman

**Affiliations:** ^1^ ICAP at Columbia University, Mailman School of Public Health, Columbia University New York NY USA; ^2^ Institute of Social and Preventive Medicine (ISPM) University of Bern Bern Switzerland; ^3^ Division of Global HIV and TB Center for Global Health CDC Atlanta GA USA; ^4^ Ministry of Health Malawi Lilongwe Malawi; ^5^ Division of Global HIV and TB Center for Global Health CDC Lesotho Maseru Lesotho; ^6^ Division of Global HIV and TB Center for Global Health CDC Zimbabwe Harare Zimbabwe; ^7^ Division of Global HIV and TB Center for Global Health CDC Malawi Lilongwe Malawi; ^8^ Division of Global HIV and TB Center for Global Health CDC Zambia Lusaka Zambia; ^9^ Ministry of Health & Child Welfare Harare Zimbabwe; ^10^ Ministry of Health Lusaka Zambia; ^11^ Research Coordination Unit Ministry of Health Maseru Lesotho; ^12^ Division of Global HIV and TB Center for Global Health CDC Eswatini Mbabane Swaziland

**Keywords:** HIV, ARV, viral suppression, 90‐90‐90 targets, second‐line ART, sub‐Saharan Africa

## Abstract

**Introduction:**

The global target for 2020 is that ≥90% of people living with HIV (PLHIV) receiving antiretroviral therapy (ART) will achieve viral load suppression (VLS). We examined VLS and its determinants among adults receiving ART for at least four months.

**Methods:**

We analysed data from the population‐based HIV impact assessment (PHIA) surveys in Eswatini, Lesotho, Malawi, Zambia and Zimbabwe (2015 to 2017). PHIA surveys are nationally representative, cross‐sectional household surveys. Data collection included structured interviews, home‐based HIV testing and laboratory testing. Blood samples from PLHIV were analysed for HIV RNA, CD4 counts and recent exposure to antiretroviral drugs (ARVs). We calculated representative estimates for the prevalence of VLS (viral load <1000 copies/mL), nonsuppressed viral load (NVL; viral load ≥1000 copies/mL), virologic failure (VF; ARVs present and viral load ≥1000 copies/mL), interrupted ART (ARVs absent and viral load ≥1000 copies/mL) and rates of switching to second‐line ART (protease inhibitors present) among PLHIV aged 15 to 59 years who participated in the PHIA surveys in Eswatini, Lesotho, Malawi, Zambia and Zimbabwe, initiated ART at least four months before the survey and were receiving ART at the time of the survey (according to self‐report or ARV testing). We calculated odds ratios and incidence rate ratios for factors associated with NVL, VF, interrupted ART, and switching to second‐line ART.

**Results:**

We included 9200 adults receiving ART of whom 88.8% had VLS and 11.2% had NVL including 8.2% who experienced VF and 3.0% who interrupted ART. Younger age, male sex, less education, suboptimal adherence, receiving nevirapine, HIV non‐disclosure, never having married and residing in Zimbabwe, Lesotho or Zambia were associated with higher odds of NVL. Among people with NVL, marriage, female sex, shorter ART duration, higher CD4 count and alcohol use were associated with lower odds for VF and higher odds for interrupted ART. Many people with VF (44.8%) had CD4 counts <200 cells/µL, but few (0.31% per year) switched to second‐line ART.

**Conclusions:**

Countries are approaching global VLS targets for adults. Treatment support, in particular for younger adults, and people with higher CD4 counts, and switching of people to protease inhibitor‐ or integrase inhibitor‐based regimens may further reduce NVL prevalence.

## 1 INTRODUCTION

The Joint United Nations Programme on HIV/AIDS (UNAIDS) has set a target for 90% of people on ART to have viral load suppression (VLS) by 2020 [1]. Maintaining VLS keeps HIV‐positive patients healthy and reduces the risk of HIV transmission [2, 3, 4, 5]. Consistent retention and adherence to an effective ART regimen are essential for maintaining VLS, but inadequate retention and adherence and use of ineffective ART regimens are common, leading to reduced individual and public health benefits of ART [6, 7, 8].

The World Health Organization (WHO) recommends routine viral load (VL) monitoring of patients receiving ART [9]. Recently, VL monitoring has been scaled up in resource‐limited settings; however, in most sub‐Saharan African countries, access remains limited. The proportion of patients who had received at least one VL test by mid‐2016 was 19% in Malawi, 11% in Côte d’Ivoire, 49% in Kenya, 43% in Namibia, 9% in Tanzania and 22% in Uganda [10]. For settings with limited VL testing capacity, WHO recommends targeted VL testing of patients with suspected treatment failure [9]. In settings without access to VL testing, healthcare workers rely on immunological or clinical criteria to detect ART failure [6]. The diagnostic accuracy of these criteria for identifying patients with virologic failure (VF) is poor, and patients may not receive adherence support, may remain on ineffective ART regimens or may be switched to second‐line ART unnecessarily [6]. Even where VL testing has been implemented, patients may not benefit from clinical management if results are not used for clinical decision‐making [6, 11].

We analysed nationally representative data from the population‐based HIV impact assessment (PHIA) surveys in Eswatini (formerly called Swaziland), Lesotho, Malawi, Zambia and Zimbabwe to examine VLS and its determinants (interrupting ART, VF and switching to second‐line ART) among adults receiving ART for at least four months.

## 2 METHODS

### 2.1 Survey design

The PHIA surveys are nationally representative, cross‐sectional household surveys. PHIA surveys use a two‐stage cluster‐based sampling design. The surveys measure HIV incidence, HIV prevalence and VLS prevalence among PLHIV and progress towards each of the UNAIDS 90‐90‐90 targets. The survey was conducted between 2015 and 2017 in the five countries included in our analysis.

Data collection included structured household and individual interviews, home‐based HIV testing using each country’s national rapid testing algorithm with immediate return of results and further laboratory testing. HIV‐positive blood specimens were tested for plasma VL. If an insufficient volume of plasma was collected HIV VL testing was performed on dried blood spots (DBS). Positive specimens were also tested for CD4 cell counts and the presence of selected antiretroviral drugs (ARVs). In each country, samples were tested for the first‐line ARVs efavirenz (EFV) and nevirapine (NVP) and the second‐line ARV lopinavir (LPV). Malawi and Zambia also tested samples for the second‐line ARV atazanavir (ATV). A qualitative high‐performance liquid chromatography/tandem mass spectrometry assay was performed to detect antiretroviral drug (ARV) in DBS. A concentration of 0.02 μg/mL was used as the cut‐off. More details of laboratory and survey methods are given in the appendix (Text S1) and in survey reports [12, 13, 14, 15, 16] which are available online at https://phia.icap.columbia.edu/resource_categories/final‐reports/.

### 2.2 Eligibility

We included HIV‐positive adults aged 15 to 59 years who participated in the five PHIA surveys, who consented to biomarker testing, who provided complete data on HIV awareness and treatment status and who were classified as receiving ART at the time of the survey either by reporting current ART use or by having detectable blood levels of selected ARVs. We excluded people who reported receiving ART for less than four months to account for people with high baseline VL who may not have had VLS at the time of data collection.

### 2.3 Measures

As per WHO definitions, we defined VLS as VL < 1000 copies/mL and NVL as VL ≥ 1000 copies/mL [9]. People with NVL were classified as either experiencing VF if any single ARV was detected in blood samples or as having interrupted ART if no ARVs were detected. We classified regimens as EFV‐based if EFV was detected, as NVP‐based if NVP was detected, and as protease inhibitor (PI)‐based if either LPV or ATV was detected. We classified non‐nucleoside reverse‐transcriptase inhibitors (NNRTI)‐based regimens as first‐line ART and PI‐based regimens as second‐line ART [9]. CD4 cell count was categorized as <100, 100 to 199, 200 to 349, 350 to 499 and ≥500 cells/µL. VL was categorized as <40, 40‐999 and ≥1000 copies/mL. DBS VL samples could not be categorized and were excluded from this analysis.

We calculated ART duration as the date of the survey interview minus the self‐reported ART start date. We categorized ART duration as <1, 1 to 2, 2 to 5, 6 to 10 and >10 years. We defined adherence to ART, based on self‐reported data, as optimal if a participant reported missing less than two doses in the last 30 days or suboptimal if the participant missed two or more doses in the last 30 days.

We used an asset‐based wealth index and categorized the wealth index scores into quintiles [17]. Households that received cash transfer, assistance for school fees, material support for education or income generation support in cash or other forms in the last 12 months were considered to have received economic support. Work for which individuals received a salary, cash or in‐kind payment in the last 12 months was classified as paid work. Alcohol use was assessed with the AUDIT‐C three‐item questionnaire. We used the cut‐off AUDIT‐C score of ≥4 in men and ≥3 in women to classify people screening positive for hazardous drinking [18, 19].

### 2.4 Statistical analysis

We estimated the prevalence of VLS and NVL among people receiving ART for at least four months and the prevalence of VF and interrupted ART among those with NVL. In a sensitivity analysis, we examined how the exclusion of people who received ART for less than four months affected NVL prevalence. We used bivariate and multivariable logistic regression to calculate odds ratios (OR) for factors associated with NVL, interrupted ART and VF. Logistic regression models accounted for stratification and clustering in the sample design. We estimated variance using the Taylor series linearization method.

We used purposeful selection of covariates to build our final regression models [20]. We considered characteristics of ART and sociodemographic and behavioural characteristics in bivariate analysis. Variables associated with the outcome at a significance level of <0.20 in bivariate analysis [21, 22] and variables that had been reported as important determinants of VLS or interruption of ART (i.e. wealth quintile, education and ART duration) [23, 24, 25, 26, 27, 28, 29] were considered in multivariable analysis. Variables not significant at the level <0.05 in multivariable analysis were successively eliminated from multivariable models starting with the least significant variable. After eliminating a variable from the model, we compared the estimates of all coefficients that remained in the smaller model to their respective estimates from the larger model and added the eliminated variable back into the model if an estimate changed by more than 20%. We checked for interactions between independent variables that remained in the final model. We imputed missing data for all variables that were considered in multivariable analysis using multiple imputation with chained equations, ran multivariable analysis on 25 imputed datasets and pooled results using Rubin’s rules [30, 31, 32, 33]. Imputation models included all variables that were considered in multivariable analysis, NVL, VF, interruption of ART, CD4 cell count, region and survey weights.

We calculated rates of switching to second‐line ART as the number of participants who switched divided by the person‐years on first‐line ART. We had no data on the timing of switching to second‐line ART but assumed that participants switched after 1.85 years, the median time from initiation of first‐line ART to switching to second‐line ART in programs in resource‐limited settings without access to routine VL monitoring [34]. We calculated incidence rate ratios (IRR) comparing switching rates by country, age, sex, CD4 count and urban or rural residence using multivariable Poisson regression. In a sensitivity analysis, we assumed that participants switched at the earliest and latest possible times (i.e. six months after initiating first‐line ART and on the day of the survey). We excluded participants with unknown ART start date and unknown ART regimen from analysis of switching rates and factors associated with switching. To ensure national population‐level representativeness of the survey, all analyses were weighted for selection probability, non‐response and non‐coverage using survey weights. We report weighted percentages and unweighted numbers of participants in all analyses if not stated otherwise. Statistical analysis was done using Stata (Version 15, Stata Corporation, College Station, TX, USA).

### 2.5 Ethical considerations

Local ethics committees and Institutional Review Boards approved the PHIA surveys. All participants provided written informed consent. Anonymized data were used for statistical analyses. More details on research ethics are given in the appendix (Text S2).

## 3 RESULTS

### 3.1 Inclusion and characteristics of participants

**Figure 1:**
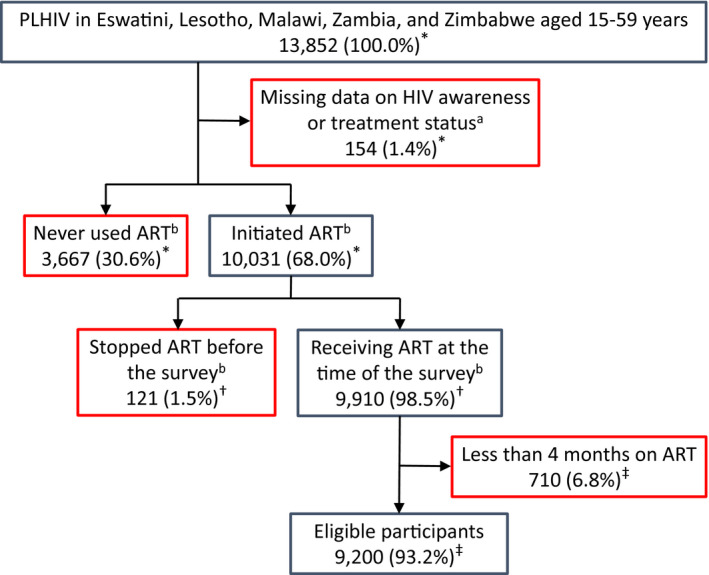
Flow diagram of inclusion of HIV‐positive adults aged 15 to 59 years on antiretroviral therapy (ART) for at least four months who participated in the Population‐based HIV Impact Assessment (PHIA) survey in Eswatini, Lesotho, Malawi, Zambia and Zimbabwe (2015 to 2017). Data are unweighted numbers of participants and weighted percentages. Participants shown in red boxes were excluded. ^a^We excluded participants with missing data on HIV awareness status, ART use, HIV RNA or ARV testing. ^b^We assessed ART use based on reported past and current ART use to treat HIV infection and ARV testing results. Participants who reported no past or current ART use and who had no detectable ARV blood levels were classified as having never used ART. Participants who reported past or current ART use or who had detectable ARV blood levels were classified as having initiated ART. Participants who reported past but no current ART use and who had no detectable ARV blood levels were classified as having ceased ART before the survey. Participants who reported receiving ART at the time of the survey, or who had detectable ARV blood levels were classified as receiving ART at the time of the survey. *Denominators for percentages are PLHIV in Eswatini, Lesotho, Malawi, Zambia and Zimbabwe aged 15 to 59 years. ^†^Denominators for percentages are participants who initiated ART. ^‡^Denominators for percentages are participants who were on ART at the time of the survey. ART, antiretroviral therapy; ARV, antiretroviral drugs; PLHIV, people living with HIV.

Out of the 13 852 adults, aged 15 to 59 years, who tested HIV‐positive during the five PHIA surveys, 10 031 (68%) had initiated ART. Of those who had initiated ART, 121 (1.5%) had stopped ART before the survey, and 9910 (98.5%) were still receiving ART at the time of the survey. Out of those still on ART at the time of the survey, we included 9200 (93.2%) participants who were on ART for at least four months (Figure 1). Most were women (64.2%) and resided in rural areas (59.5%). The median age was 39 years (interquartile range [IQR], 32 to 46 years). Most (61.5%) were married or cohabitating. Few (6.9%) reported being unaware of their HIV‐positive status or being HIV negative. Most (69.0%) had disclosed their HIV status to a family member (Table 1, Table S1). The prevalence of participants who screened positive for problematic or hazardous drinking or active alcohol use disorder was 8.3% (Table S2).

**Table 1:** Sociodemographic characteristics of HIV‐positive adults receiving antiretroviral therapy in Lesotho, Malawi, Eswatini, Zambia and Zimbabwe (2015 to 2017)

Characteristic	Lesotho	Malawi	Eswatini	Zambia	Zimbabwe	Total
	N = 2177	N = 1406	N = 2025	N = 1424	N = 2168	N = 9200
Residence, n (%)	2177 (100.0)	1406 (100.0)	2025 (100.0)	1424 (100.0)	2168 (100.0)	9200 (100.0)
Urban	991 (49.8)	644 (24.9)	487 (29.1)	868 (62.3)	656 (35.8)	3646 (40.5)
Rural	1186 (50.2)	762 (75.1)	1538 (70.9)	556 (37.7)	1512 (64.2)	5554 (59.5)
Sex, n (%)	2177 (100.0)	1406 (100.0)	2025 (100.0)	1424 (100.0)	2168 (100.0)	9200 (100.0)
Male	646 (38.0)	401 (35.6)	587 (30.3)	414 (35.8)	660 (36.3)	2708 (35.8)
Female	1531 (62.0)	1005 (64.4)	1438 (69.7)	1010 (64.2)	1508 (63.7)	6492 (64.2)
Age in years, n (%)	2177 (100.0)	1406 (100.0)	2025 (100.0)	1424 (100.0)	2168 (100.0)	9200 (100.0)
15 to 19	56 (2.7)	22 (2.0)	70 (3.3)	29 (2.6)	83 (4.6)	260 (3.1)
20 to 24	116 (4.7)	76 (4.3)	124 (6.1)	65 (4.9)	85 (4.4)	466 (4.6)
25 to 44	1293 (61.6)	923 (65.0)	1294 (67.0)	895 (62.2)	1287 (61.4)	5692 (62.9)
45 to 59	712 (30.9)	385 (28.7)	537 (23.6)	435 (30.3)	713 (29.7)	2782 (29.3)
Median (IQR)	39 (32 to −47)	39 (32 to 45)	37 (30 to 44)	40 (33 to 46)	39 (32 to 46)	39 (32 to 46)
Wealth quintile, n (%)	2174 (100.0)	1406 (100.0)	2024 (100.0)	1413 (100.0)	2168 (100.0)	9185 (100.0)
Lowest	440 (17.4)	147 (14.1)	512 (23.1)	119 (8.2)	554 (21.1)	1772 (15.8)
Second	493 (21.3)	172 (17.0)	457 (21.7)	157 (10.8)	436 (18.6)	1715 (16.7)
Middle	480 (22.5)	193 (18.3)	429 (20.7)	295 (20.2)	399 (19.2)	1796 (19.6)
Fourth	400 (20.0)	287 (23.4)	349 (19.2)	371 (26.0)	393 (21.7)	1800 (22.9)
Highest	361 (18.8)	607 (27.1)	277 (15.3)	471 (34.8)	386 (19.3)	2102 (24.9)
Education, n (%)	2176 (100.0)	1405 (100.0)	2022 (100.0)	1424 (100.0)	2168 (100.0)	9195 (100.0)
No or primary	1309 (59.1)	999 (77.5)	890 (41.3)	649 (44.0)	891 (36.9)	4738 (51.7)
Secondary or higher	867 (40.9)	406 (22.5)	1132 (58.7)	775 (56.0)	1277 (63.1)	4457 (48.3)
Marital status, n (%)	2171 (100.0)	1404 (100.0)	2012 (100.0)	1420 (100.0)	2163 (100.0)	9170 (100.0)
Never married	330 (15.5)	76 (4.6)	654 (33.0)	133 (10.2)	208 (10.0)	1401 (10.6)
Married/living together	1130 (54.4)	909 (65.9)	1019 (50.5)	873 (63.1)	1287 (61.0)	5218 (61.5)
Divorced, separated or widowed	711 (30.1)	419 (29.5)	339 (16.5)	414 (26.7)	668 (29.0)	2551 (27.9)
Economic support, n (%)	2177 (100.0)	1406 (100.0)	2025 (100.0)	1424 (100.0)	2168 (100.0)	9200 (100.0)
No	1720 (80.4)	1302 (91.1)	1204 (60.7)	1364 (95.8)	1871 (87.1)	7461 (88.1)
Yes	457 (19.6)	104 (8.9)	821 (39.3)	60 (4.2)	297 (12.9)	1739 (11.9)
Paid work, n (%)	2177 (100.0)	1406 (100.0)	2025 (100.0)	1421 (100.0)	2167 (100.0)	9196 (100.0)
Yes	871 (43.9)	480 (32.8)	936 (48.5)	537 (40.2)	763 (37.4)	3587 (38.2)
No	1306 (56.1)	926 (67.2)	1089 (51.5)	884 (59.8)	1404 (62.6)	5609 (61.8)
Disclosure to a family member, n (%)	2177 (100.0)	1406 (100.0)	2025 (100.0)	1424 (100.0)	2168 (100.0)	9200 (100.0)
No	540 (25.9)	344 (24.2)	660 (32.6)	291 (20.5)	507 (24.9)	2342 (24.2)
Yes	1525 (68.1)	977 (69.4)	1307 (64.1)	1005 (70.1)	1544 (68.8)	6358 (69.0)
Unknown (unaware of HIV status)	112 (5.9)	85 (6.4)	58 (3.3)	128 (9.3)	117 (6.3)	500 (6.9)

IQR, interquartile range; N, Number of eligible participants; n, number of participants providing a valid response (weighted %).

### 3.2 Characteristics of and response to ART

Median duration on ART was reported as four years (IQR 2 to 7 years). Based on ARV testing, most participants (91.3%) received an EFV‐based first‐line regimen and 90.6% reported optimal adherence. The proportion of participants who received second‐line regimens ranged from 0.3% in Zimbabwe to 3.4% in Eswatini. The median CD4 count was 467 cells/µL (IQR 315 to 641 cells/µL). Overall, 20.5% (1837/8972) of participants who received a plasma viral load test had a detectable VL of ≥40 copies/mL. The prevalence of VLS was 88.8% (n = 8236), 11.2% (n = 964) had NVL including 8.2% (n = 686) who had evidence of VF and 3.0% (n = 278) of interrupted ART (Table 2). In the sensitivity analysis including of participants who received ART for less than four months, NVL prevalence was 12.4% (298/2416) in Lesotho, 8.8% (131/1498) in Malawi, 9.0% in Swaziland (193/2175), 10.8% (161/1542) in Zambia, 15.1% (314/2279) in Zimbabwe and 11.7% (1097/9910) across all five countries.

**Table 2:** Characteristics of and response to antiretroviral therapy (ART) among HIV‐positive adults in Lesotho, Malawi, Eswatini, Zambia and Zimbabwe (2015 to 2017)

	Lesotho	Malawi	Eswatini	Zambia	Zimbabwe	Total
	N = 2177	N = 1406	N = 2025	N = 1424	N = 2168	N = 9200
Duration on ART, n (%)	2177 (100.0)	1406 (100.0)	2025 (100.0)	1424 (100.0)	2168 (100.0)	9200 (100.0)
4 to 12 months	336 (15.6)	144 (10.2)	251 (12.8)	169 (12.1)	216 (10.7)	1116 (11.5)
1 to 2 years	285 (13.3)	188 (13.3)	283 (13.8)	186 (13.5)	275 (12.4)	1217 (13.1)
2 to 5 years	574 (25.9)	434 (30.8)	613 (30.0)	383 (26.3)	734 (33.6)	2738 (30.1)
5 to 10 years	630 (28.3)	417 (29.4)	609 (29.9)	415 (28.1)	702 (31.2)	2773 (29.6)
>10 years	153 (7.0)	104 (7.1)	162 (7.5)	108 (7.9)	81 (3.8)	608 (6.2)
Missing	199 (9.9)	119 (9.3)	107 (5.9)	163 (12.0)	160 (8.3)	748 (9.5)
Median (IQR)	4 (1 to 7)	4 (2 to 7)	4 (2 to 7)	4 (2 to 7)	4 (2 to 6)	4 (2 to 7)
ART regimen, n (%)	2076 (100.0)	1357 (100.0)	1956 (100.0)	1365 (100.0)	2049 (100.0)	8803 (100.0)
EFV‐based	1750 (84.7)	1257 (93.1)	1477 (76.2)	1223 (89.3)	1970 (96.3)	7677 (91.3)
NVP‐based	303 (14.1)	87 (6.2)	412 (20.4)	98 (7.4)	74 (3.4)	974 (7.2)
LPV‐based	23 (1.2)	0 (0.0)	67 (3.4)	42 (3.1)	5 (0.3)	137 (1.2)
ATV‐based	NA	13 (0.7)	NA	2 (0.1)	NA	15 (0.2)
Adherence, n (%)	2022 (100.0)	1299 (100.0)	1881 (100.0)	1266 (100.0)	2019 (100.0)	8487 (100.0)
Optimal	1769 (87.1)	1142 (87.4)	1692 (90.1)	1154 (90.7)	1908 (94.2)	7665 (90.6)
Suboptimal	253 (12.9)	157 (12.6)	189 (9.9)	112 (9.3)	111 (5.8)	822 (9.4)
CD4 count per µL, n (%)	2177 (100.0)	1405 (100.0)	2024 (100.0)	1420 (100.0)	2166 (100.0)	9192 (100.0)
<100	52 (2.6)	34 (2.4)	25 (1.3)	35 (2.6)	74 (3.9)	220 (2.9)
100 to 199	120 (6.1)	91 (7.5)	68 (3.5)	105 (7.9)	177 (9.4)	561 (7.8)
200 to 349	321 (15.4)	236 (16.4)	249 (12.5)	273 (19.5)	503 (23.7)	1582 (19.3)
350 to 499	462 (21.9)	342 (24.5)	436 (21.5)	405 (28.1)	573 (25.2)	2218 (25.2)
≥500	1222 (54.0)	702 (49.2)	1246 (61.2)	602 (41.9)	839 (37.8)	4611 (44.8)
Median (IQR)	528 (360 to 701)	497 (338 to 652)	571 (409 to 769)	457 (311 to 622)	422 (278 to 591)	467 (315 to 641)
Viral load in copies/mL, n (%)^a^	2099 (100.0)	1399 (100.0)	1973 (100.0)	1343 (100.0)	2158 (100.0)	8972 (100.0)
<40	1627 (77.2)	1212 (85.7)	1577 (79.3)	1055 (78.6)	1664 (75.8)	7135 (79.5)
40 to 999	238 (11.6)	69 (5.6)	232 (12.3)	153 (11.1)	206 (9.6)	898 (9.2)
≥1000	234 (11.2)	118 (8.7)	164 (8.4)	135 (10.3)	288 (14.6)	939 (11.3)
ART outcome, n (%)	2177 (100.0)	1406 (100.0)	2025 (100.0)	1424 (100.0)	2168 (100.0)	9200 (100.0)
Viral load suppression	1929 (88.5)	1287 (91.3)	1858 (91.7)	1285 (90.1)	1877 (85.3)	8236 (88.8)
Nonsuppressed viral load	248 (11.5)	119 (8.7)	167 (8.3)	139 (9.9)	291 (14.7)	964 (11.2)
Virologic failure	174 (8.0)	75 (5.8)	116 (5.7)	99 (7.2)	222 (11.5)	686 (8.2)
Interrupted ART	74 (3.5)	44 (2.9)	51 (2.6)	40 (2.8)	69 (3.2)	278 (3.0)

Vial load suppression: viral load < 1000 copies/mL; nonsuppressed viral load: viral load ≥ 1000 copies/mL. Virologic failure: nonsuppressed viral load and antiretroviral drugs detected; interrupted ART: nonsuppressed viral load and no antiretroviral drugs detected. Adherence was defined as optimal if a participant reported missing less than two doses of ART in the last 30 days and suboptimal if a participant reported missing two or more doses in the last 30 days. N, number of eligible participants; n, number of participants providing a valid response (weighted %). IQR, interquartile range; ART, antiretroviral therapy; EFV, efavirenz; NVP, nevirapine; LPV, lopinavir; ATV, atazanavir.

^a^ Dried blood spot viral load test results (n = 228) could not be classified and were excluded.

### 3.3 Characteristics of participants with NVL

Table 3 shows the characteristics of participants with NVL. One‐quarter of participants with NVL (26.7%) had evidence of interrupted ART, and three‐quarters (73.3%) were experiencing VF. Most participants with NVL (59.8%) were women. Median age of participants with NVL was 36 years (IQR, 29 to 43 years). Median CD4 count among participants with VF was 222 cells/µL (IQR 116 to 381 cells/µL), and median duration on ART was 4.5 years (IQR 2.6 to 6.9). Only 1.8% of participants with VF received second‐line ART.

**Table 3:** Characteristics of adults on antiretroviral therapy with nonsuppressed viral load: Results from the Lesotho, Malawi, Eswatini, Zambia and Zimbabwe Population‐based HIV Impact Assessment survey (2015 to 2017)

	Interrupted ART	Virologic failure	Nonsuppressed viral load
	N = 278 [26.7%]	N = 686 [73.3%]	N = 964 [100.0%]
Sex, n (%)	278 (100.0)	686 (100.0)	964 (100.0)
Male	68 (28.9)	246 (44.3)	314 (40.2)
Female	210 (71.1)	440 (55.7)	650 (59.8)
Age in years, n (%)	278 (100.0)	686 (100.0)	964 (100.0)
15 to 19	19 (6.8)	45 (5.7)	64 (6.0)
20 to 24	36 (10.8)	58 (7.4)	94 (8.3)
25 to 44	184 (66.4)	439 (66.5)	623 (66.5)
45 to 59	39 (15.9)	144 (20.4)	183 (19.2)
Median (IQR)	34 (27 to 41)	37 (30 to 43)	36 (29 to 43)
Marital status, n (%)	277 (100.0)	681 (100.0)	958 (100.0)
Never married	60 (13.1)	148 (17.5)	208 (16.4)
Married or living together	149 (56.6)	360 (60.5)	509 (59.5)
Divorced, separated or widowed	68 (30.2)	173 (21.9)	241 (24.2)
AUDIT‐C score, n (%)	202 (100.0)	506 (100.0)	708 (100.0)
0	171 (82.4)	417 (81.5)	588 (81.7)
1 to 2	13 (6.9)	43 (9.3)	56 (8.7)
3 to 4	5 (3.7)	26 (4.9)	31 (4.6)
5 to 8	9 (4.0)	18 (4.0)	27 (4.0)
9 to 12	4 (3.0)	2 (0.3)	6 (1.0)
Antiretroviral drug detected, n (%)	278 (100.0)	686 (100.0)	964 (100.0)
EFV	0 (0.0)	567 (87.6)	567 (64.2)
NVP	0 (0.0)	105 (10.7)	105 (7.8)
LPV	0 (0.0)	12 (1.4)	12 (1.0)
ATV	0 (0.0)	2 (0.4)	2 (0.3)
No ARVs detected	278 (100.0)	0 (0.0)	278 (26.7)
Duration on ART, n (%)	271 (100.0)	564 (100.0)	835 (100.0)
4 to 12 months	58 (18.7)	53 (10.1)	111 (12.7)
1 to 2 years	53 (19.9)	65 (10.1)	118 (13.1)
2 to 5 years	83 (30.4)	191 (34.8)	274 (33.5)
5 to 10 years	59 (23.1)	204 (36.4)	263 (32.4)
>10 years	18 (8.0)	51 (8.6)	69 (8.4)
Median (IQR)	2.9 (1.2 to 5.8)	4.5 (2.6 to 6.9)	4.2 (1.9 to 6.6)
CD4 cell count per µL, n (%)	276 (100.0)	685 (100.0)	961 (100.0)
<100	33 (12.7)	128 (21.7)	161 (19.3)
100 to 199	52 (21.2)	139 (23.1)	191 (22.6)
200 to 349	68 (22.0)	170 (25.7)	238 (24.7)
350 to 499	61 (21.8)	129 (16.4)	190 (17.8)
≥500	62 (22.4)	119 (13.2)	181 (15.6)
Median (IQR)	300 (165 to 497)	222 (116 to 381)	237 (126 to 414)
Viral load log10 copies/mL, n (%)			
3 to 4	59 (20.2)	254 (35.9)	313 (31.7)
4 to 5	152 (56.0)	299 (44.0)	451 (47.2)
5 to 6	61 (22.0)	125 (19.1)	186 (19.9)
≥6	6 (1.9)	8 (1.0)	14 (1.2)
Median (IQR)	4.5 (4.2 to 5.0)	4.3 (3.8 to 4.8)	4.4 (3.8 to 4.9)

Nonsuppressed viral load was defined as viral load ≥1000 copies/mL. ART, antiretroviral therapy; EFV, efavirenz; IQR, interquartile range; LPV, lopinavir: ATV, atazanavir; N, number of eligible participants; n, number of participants providing a valid response (weighted col %) [weighted row %]; NVP, nevirapine.

### 3.4 Factors associated with NVL

The adjusted odds ratios (aOR) for NVL for participants in Zimbabwe (aOR 2.61; 95% confidence interval [CI]: 2.03 to 3.35), Lesotho (aOR 1.61; 95% CI: 1.26 to 2.05), and Zambia (aOR 1.52; 95% CI: 1.14 to 2.02) were higher than in Eswatini. The odds for NVL in people aged 15 to 24 years were 2 to 3 times higher than in those aged 45 to 59 years. The odds of NVL were slightly increased in men (aOR 1.28; 95% CI: 1.05 to 1.55), in participants with low educational levels (aOR 1.27; 95% CI: 1.06 to 1.52), in those who had never married (aOR 1.42; 95% CI: 1.02 to 1.98) and in those who had not disclosed their HIV status to a family member (aOR 1.32; 95% CI: 1.06 to 1.66). Participants receiving NVP‐based ART had higher odds for NVL (aOR 1.84; 95% CI: 1.34 to 2.51) than those receiving EFV‐based regimens. Odds for NVL were also higher in participants who reported sub‐optimal adherence (aOR 1.81; 95% CI: 1.37 to 2.39; Table 4).

**Table 4:** Factors associated with nonsuppressed viral load among HIV‐positive participants in the Population‐based HIV Impact Assessment survey in Eswatini, Lesotho, Malawi, Zambia and Zimbabwe (2015 to 2017)

	Nonsuppressed viral load			
	Bivariate analysis		Multivariable analysis	
	OR (95% CI)	P‐value	aOR (95% CI)	P‐value
Characteristics of participants				
Country of residence		<0.001		<0.001
Lesotho	1.43 (1.14 to 1.79)		1.61 (1.26 to 2.05)	
Malawi	1.05 (0.78 to 1.44)		1.25 (0.90 to 1.74)	
Eswatini	1.00		1.00	
Zambia	1.22 (0.94 to 1.57)		1.52 (1.14 to 2.02)	
Zimbabwe	1.90 (1.53 to 2.35)		2.61 (2.03 to 3.35)	
Age, years		<0.001		<0.001
15 to 19	3.47 (2.23 to 5.41)		2.15 (1.23 to 3.78)	
20 to 24	3.17 (2.17 to 4.62)		3.13 (2.08 to 4.71)	
25 to 44	1.70 (1.34 to 2.14)		1.81 (1.42 to 2.32)	
45 to 59	1.00		1.00	
Sex		0.022		0.013
Male	1.24 (1.03 to 1.48)		1.28 (1.05 to 1.55)	
Female	1.00		1.00	
Marital status		<0.001		0.110
Never married	1.72 (1.33 to 2.23)		1.42 (1.02 to 1.98)	
Married or living together	1.00		1.00	
Divorced, separated or widowed	0.89 (0.72 to 1.09)		1.02 (0.82 to 1.27)	
Education		0.977		0.010
No or primary	1.00 (0.83 to 1.20)		1.27 (1.06 to 1.52)	
Secondary or higher	1.00		1.00	
Disclosure to a family member		0.003		0.014
No	1.29 (1.04 to 1.61)		1.32 (1.06 to 1.66)	
Yes	1.00		1.00	
Not self‐reported positive	1.64 (1.16 to 2.32)		1.39 (0.96 to 2.02)	
Characteristics of antiretroviral therapy				
Antiretroviral regimen		0.003		0.001
EFV‐based	1.00		1.00	
NVP‐based	1.66 (1.23 to 2.24)		1.84 (1.34 to 2.51)	
PI‐based	1.65 (0.80 to 3.39)		1.86 (0.88 to 3.89)	
Adherence		<0.001		<0.001
Optimal	1.00		1.00	
Suboptimal	1.91 (1.46 to 2.50)		1.81 (1.37 to 2.39)	

Multivariable analysis adjusted for country, age, sex, marital status, education, disclosure of HIV status to a family member, antiretroviral regimen and adherence. Nonsuppressed viral load was defined as viral load ≥1000 copies/mL. P‐values of categorical variables are for joint test for significance. aOR, adjusted odds ratios; ART, antiretroviral therapy; EFV, efavirenz; NVP, nevirapine; OR, odds ratio; PI, protease inhibitor.

### 3.5 Factors associated with VF and interrupted ART among participants with NVL

Table 5 shows aORs comparing the odds for VF among participants with NVL. The adjusted odds for VF among participants aged 15 to 19 years were lower (aOR 0.32; 95% CI: 0.14 to 0.76) than in those aged 44 to 59 years. Women had almost half the odds (aOR 0.54; 95% CI: 0.29 to 0.99) of VF compared to men. Married (aOR 0.53; 95% CI: 0.31 to 0.93) and divorced participants (aOR 0.37; 95% CI: 0.19 to 0.71) had lower odds for VF than participants who never married. The odds for VF were 66% (aOR 0.34; 95% CI: 0.17 to 0.71) and 68% (aOR 0.32; 95% CI: 0.17 to 0.59) lower in participants receiving ART for <1 year and one to two years, respectively, compared to those receiving ART for five to ten years. Participants with a CD4 count of 350 to 500 cells/µL (aOR 0.48 95% CI: 0.27 to 0.85) and those with a CD4 count ≥500 cells/µL (aOR 0.40 95% CI: 0.19 to 0.81) had lower odds of VF than those with a CD4 cell count <100 cells/µL. Factors associated with VF were inversely associated with interrupted ART because participants with NVL either had VF or interrupted ART.

**Table 5:** Factors associated with virologic failure among participants with nonsuppressed viral load in Eswatini, Lesotho, Malawi, Zambia and Zimbabwe (2015 to 2017)

	Virologic failure			
	Bivariate analysis		Multivariable analysis	
	OR (95% CI)	P‐value	aOR (95% CI)	P‐value
Sociodemographic characteristics				
Age in years		0.250		0.056
15 to 19	0.66 (0.30 to 1.47)		0.32 (0.14 to 0.76)	
20 to 24	0.53 (0.28 to 1.01)		0.54 (0.24 to 1.18)	
25 to 44	0.78 (0.49 to 1.23)		0.89 (0.56 to 1.42)	
45 to 59	1.00		1.00	
Sex		0.002		0.047
Male	1.00		1.00	
Female	0.51 (0.34 to 0.78)		0.54 (0.29 to 0.99)	
Marital status		0.043		0.012
Never married	1.00		1.00	
Married or living together	0.80 (0.53 to 1.20)		0.53 (0.31 to 0.93)	
Divorced, separated or widowed	0.54 (0.34 to 0.88)		0.37 (0.19 to 0.7)	
Antiretroviral therapy characteristics				
Duration on ART in years		0.001		<0.001
<1	0.39 (0.20 to 0.76)		0.34 (0.17 to 0.71)	
1 to 2	0.37 (0.21 to 0.65)		0.32 (0.17 to 0.59)	
2 to 5	0.74 (0.47 to 1.17)		0.75 (0.45 to 1.24)	
5 to 10	1.00		1.00	
>10	0.69 (0.33 to 1.40)		0.62 (0.30 to 1.26)	
CD4 cell count per µL		0.004		0.048
<100	1.00		1.00	
100 to 199	0.65 (0.40 to 1.05)		0.59 (0.37 to 0.95)	
200 to 349	0.69 (0.38 to 1.25)		0.67 (0.37 to 1.21)	
350 to 499	0.44 (0.26 to 0.76)		0.48 (0.27 to 0.85)	
≥500	0.35 (0.19 to 0.63)		0.40 (0.19 to 0.81)	
Behavioural characteristics				
AUDIT‐C score		<0.001		0.001
0	1.00		1.00	
1 to 2	1.33 (0.67 to 2.63)		1.01 (0.41 to 2.47)	
3 to 4	1.26 (0.76 to 2.11)		0.93 (0.46 to 1.87)	
5 to 8	1.00 (0.55 to 1.81)		0.55 (0.26 to 1.20)	
9 to 12	0.10 (0.04 to 0.28)		0.09 (0.03 to 0.28)	

Nonsuppressed viral load was defined as a viral load of ≥1000 copies/mL. Multivariable analysis adjusted for all variables shown in the table. P‐values of categorical variables are for joint test for significance. aOR, adjusted odds ratios; ART, antiretroviral therapy; ARV, antiretroviral medication; OR, odds ratio.

### 3.6 Rates of and factors associated with switching to second‐line ART

Overall, 131 (1.4%) of 8066 participants with known date of ART initiation and known regimen switched to second‐line ART, for an annual switching rate of 0.31% (95% CI: 0.25 to 0.40). Annual switching rates were 0.22% (95% CI: 0.14 to 0.36) in Lesotho, 0.15% (95% CI: 0.08 to 0.35) in Malawi, 0.72% (95% CI: 0.54 to 0.96) in Eswatini, 0.73% (95% CI: 0.52 to 1.04) in Zambia and 0.06% (95% CI: 0.02 to 0.33) in Zimbabwe. Compared to Eswatini, adjusted switching rates were more than 10 times lower in Zimbabwe (IRR, 0.07; 95% CI: 0.02 to 0.24), five times lower in Malawi (IRR 0.19; 95% CI: 0.09 to 0.44), more than three times lower in Lesotho (IRR 0.28; 95% CI: 0.15 to 0.52) and similar in Zambia (IRR 0.86; 95% CI: 0.57 to 1.32).

People with CD4 counts <100 cells/µL were more than three times (IRR 3.52; 95% CI: 1.05 to 11.76) and those with CD4 counts of 100 to 199 cells/µL were more than two times (IRR 2.29; 95% CI: 1.05 to 5.01) as likely to switch to second‐line ART than those with CD4 count of ≥500 cells/µL. Age, sex and residence (rural or urban) were not associated with switching rates.

Analysis of switching rates was not sensitive to assumptions on the timing of switching. The overall annual switching rate remained 0.31% whether we assumed that participants switched six months after initiation of first‐line ART or on the day of the survey (Table S3).

## 4 DISCUSSION

These population‐based nationally representative surveys showed that these five southern African countries are approaching UNAIDS VLS targets despite limited access to VL monitoring. We found that 88.8% of adults on ART achieved VLS (VL < 1000 copies/mL), while 11.2% had NVL (VL ≥ 1000 copies/mL). Sociodemographic characteristics, current ART regimen, adherence and HIV status disclosure were associated with NVL. One‐quarter of participants with NVL had no detectable blood concentrations of ARVs and were classified as having interrupted ART, while three‐quarters of participants with NVL had evidence of recent ARV exposure and were classified as experiencing VF. Marriage, female sex, shorter ART duration, higher CD4 count and alcohol use were associated with higher odds for interrupted ART and lower odds for VF. Almost half of the people with VF were severely immunosuppressed although they received ART for several years. Switching rates varied considerably between countries, but even in countries with the highest switching rates, very few patients had switched to second‐line therapy.

Low switching rates, as documented in this study, indicate substantial gaps in monitoring of ART and/or clinical management of patients with treatment failure. HIV cohort studies from low‐income and middle‐income countries showed that each year about 3% of adults have a confirmed virologic treatment failure of first‐line ART and require switching to second‐line ART [6, 35]. We found nationally representative switching rates far below the annual failure rate of 3%: annual switching rates in our study ranged from 0.06% to 0.73%. Limited access to VL monitoring likely explains these low switching rates. When PHIA data were collected, the five countries mainly relied on CD4 monitoring and clinical monitoring with limited access to targeted VL testing [10, 36, 37]. Without routine VL monitoring, treatment failure often remains undetected and patients are not switched to second‐line ART, or patients are switched late and at low CD4 cell counts [6]. Our study demonstrates the limitations of clinical and immunological monitoring of ART and supports country‐level efforts to scale‐up VL testing for early detection of NVL.

Once patients with NVL have been identified, healthcare providers need to distinguish between patients who require adherence support and those who need a new ART regimen. We noted that more than one‐quarter of participants with NVL tested negative for recent exposure to ARVs, indicating that temporary interruption of ART was the likely reason for NVL in these individuals. Young women, those with high CD4 cell counts, and those who were on ART for <2 years were more likely to interrupt ART. Suboptimal retention and adherence among women who started ART during pregnancy under Option B+ guidelines which recommend rapid ART initiation for pregnant and breastfeeding women may explain these findings [38, 39, 40]. In line with previous data, our study suggests that a substantial proportion of people with NVL interrupted ART and may benefit from adherence interventions. The ANRS 12110 trial showed that one‐third of patients with NVL had no viral resistance [41]. The DART trial showed that one‐third of patients with NVL re‐suppressed without changing their ART regimen [42]. A systematic review of five observational studies from South Africa, Eswatini, Thailand and France showed that more than 70% of patients with NVL re‐suppressed following adherence intervention [43]. In agreement with earlier reports, our study supports WHO guidelines to offer adherence support and confirmatory VL testing to patients with a first NVL to avoid unnecessary switching of ART [9]. Our study also underlines the importance of adherence support for patients who start ART with high CD4 cell counts, especially during the initial phase of treatment.

Optimizing ART regimens likely can support countries’ efforts to increase VLS rates among people receiving ART. In December 2018, WHO recommended dolutegravir (DTG), tenofovir (TDF), and either lamivudine or emtricitabine (XTC) as the preferred first‐line regimen, and low‐ and middle‐income countries are transitioning from the current first‐line regimen TDF/XTC/EFV to the new DTG‐based regimen [44]. DTG is safer and has a higher genetic barrier to resistance than EFV. Data from randomized controlled trials support switching people with undetectable VL from an EFV‐based regimen to a DTG‐based regimen; however, currently, no evidence supports this switch for those with detectable or unknown VL [45]. Our finding that about 20% of people receiving ART had a detectable VL > 40 copies/mL underlines the need for data to support switching from a failing current first‐line treatment to effective second‐line treatments. However, the effectiveness of switching from TDF/XTC/EFV to TDF/XTC/DTG needs to be evaluated. Our data suggest that men, individuals who never married, and those with a poor immunologic response to long‐term ART are at increased risk of VF and should be monitored carefully after switching to DTG.

Our study has several strengths and limitations. The national representativeness of our findings and the richness of collected sociodemographic, behavioural and laboratory data including measures on viral suppression and ARV detection are important strengths of our study. Our findings have to be considered in the view of several limitations. Our definition of VF did not rely on drug resistance data, and our ability to distinguish between patients with NVL due to inadequate adherence, drug resistance or both was limited. We classified participants as having interrupted or failing ART based on self‐report of current ART use and laboratory testing for detectable concentrations of selected ARVs. Both data sources have limitations that may lead to misclassification. Self‐reported data on ART might be susceptible to recall and social desirability bias. Likewise, laboratory errors could result in misclassification. We also acknowledge that our estimates for the prevalence of participants who interrupted ART do not include individuals who stopped ART before the survey. In our study, only 1.5% of the participants with a history of ART use reported having stopped ART before the survey. HIV cohort studies found higher rates of treatment discontinuation [8, 46, 47, 48]. PHIA estimates for treatment discontinuation might be an underestimate because PLHIV who stopped ART might underreport prior ART use or awareness of their own HIV status. The PHIA survey only included people who were alive. High mortality among individuals who remain on failing first‐line regimens might partially explain the low NVL prevalence among those who survived and were included in the survey [6, 49]. Finally, the rates of switching to second‐line ART in Lesotho, Eswatini and Zimbabwe may be underestimates because samples from these countries were tested for LPV but not ATV, although both drugs are WHO‐recommended second‐line treatments [9]. However, the ARVs tested were selected according to national treatment guidelines, and we assumed very few people in Lesotho, Eswatini and Zimbabwe would have received ATV.

## 5 CONCLUSIONS

The five countries included in our analysis have low rates of NVL and are approaching UNAIDS VLS targets, despite limited access to VL monitoring and possible suboptimal clinical management of patients with NVL. Treatment support, in particular for younger adults, and people with higher CD4 counts, and switching of patients to PI‐ or DTG‐based regimens may further reduce NVL prevalence.

## COMPETING INTERESTS

The authors have no conflicts of interest to declare.

## AUTHORS’ CONTRIBUTIONS

AH, ER and JJ conceptualized the study. AH did statistical analysis and wrote the first draft of the manuscript which was revised by ER and JJ. All authors contributed to interpretation of data and provided critical inputs in the draft manuscript. ER, HA, AJH, NP, SJ, SS, AL, HP, AS, JR, KF, EK, GB, DW, BP, KS, DB, TK, SB, GM, OM, BB, KS, LM, KT, TA, KB, AV and JJ contributed to design and implementation of PHIA surveys. All authors have read and approved the final manuscript.

## Supporting information


**Text S1.** Laboratory methods for processing of HIV‐positive specimens
**Text S2.** Ethical oversight over the Population‐based HIV Impact Assessment (PHIA) surveys in participating countries.
**Table S1.** Characteristics of HIV status disclosure among adults on antiretroviral therapy who participated in the Population‐based HIV Impact Assessment (PHIA) survey in Lesotho, Malawi, Eswatini, Zambia and Zimbabwe (2015 to 2017)
**Table S2.** Prevalence of alcohol use disorder among adults on antiretroviral therapy who participated in the Population‐based HIV Impact Assessment (PHIA) survey in Malawi, Eswatini, Zambia, and Zimbabwe (2015 to 2017)
**Table S3.** Sensitivity analysis of rates of switching to second‐line ART among HIV‐positive adults who participated in the Population‐based HIV Impact Assessment (PHIA) survey in Lesotho, Malawi, Eswatini, Zambia, and Zimbabwe (2015 to 2017)
