# Supplementary material for: Prevalence of nonsuppressed viral load and associated factors among HIV‐positive adults receiving antiretroviral therapy in Eswatini, Lesotho, Malawi, Zambia and Zimbabwe (2015 to 2017): results from population‐based nationally representative surveys
Source: J Int AIDS Soc. 2020 Nov 22;23(11):e25631. doi: 10.1002/jia2.25631 (PMC7680921; doi:10.1002/jia2.25631)
Supplement: Supplementary file 1 — Text S1. Laboratory methods for processing of HIV‐positive specimens Text S2. Ethical oversight over the Population‐based HIV Impact Assessment (PHIA) surveys in participating countries. Table S1. Characteristics of HIV status disclosure among adults on antiretroviral therapy who participated in the Population‐based HIV Impact Assessment (PHIA) survey in Lesotho, Malawi, Eswatini, Zambia and Zimbabwe (2015 to 2017) Table S2. Prevalence of alcohol use disorder among adults on antiretroviral therapy who participated in the Population‐based HIV Impact Assessment (PHIA) survey in Malawi, Eswatini, Zambia, and Zimbabwe (2015 to 2017) Table S3. Sensitivity analysis of rates of switching to second‐line ART among HIV‐positive adults who participated in the Population‐based HIV Impact Assessment (PHIA) survey in Lesotho, Malawi, Eswatini, Zambia, and Zimbabwe (2015 to 2017) [file JIA2-23-e25631-s001.docx]

**Supplementary appendix**

**Text S1: Laboratory methods for processing of HIV-positive specimens**

All HIV-positive results were laboratory-confirmed using Bio-Rad Geenius HIV1/2 (Bio-Rad, Hercules, CA). People who tested HIV-seropositive received PIMA point-of-care CD4 testing (Alere, Waltham, MA) with immediate return of results and submitted plasma specimens for HIV-1 RNA VL testing using the Abbott m2000 System (Abbott Molecular Inc., Chicago, IL) or the Roche COBAS AmpliPrep/COBAS TaqMan platform (Roche Molecular Diagnostics, Pleasanton, CA). The same platforms were used to test dried blood spots (DBS) for VL, in addition to the NucliSENSTM EasyQ HIV-1 v2.0 assay on the bioMérieux (Marcy-l’Étoile, France) for Zimbabwe.

A qualitative high-performance liquid chromatography/tandem mass spectrometry assay was performed to detect antiretroviral drug (ARV) in DBS at the Division of Clinical Pharmacology, University of Cape Town. The samples were processed with a protein precipitation extraction method. Deuterated internal standards were used for each analyte. The extraction procedure was followed by liquid chromatographic separation using a Phenomenex Kinetex EVO C18 (Phenomenex Helvetia, Basel, Switzerland) (1.7 μm; 2.1 × 50 mm; 100 Å) analytical column. An AB Sciex API 4000 mass spectrometer (SCIEX, Framingham, MA, USA) at unit resolution in the multiple reaction monitoring mode was used to monitor the transition of the protonated precursor ions m/z 705.6, 316.0, 629.6, and 267.1 to the product ions m/z 168.2, 243.9, 447.3, and 226.0 for atazanavir, efavirenz, lopinavir, and nevirapine respectively. Electro Spray ionisation was used for ion production. The assay was validated over the range of 0.02–5.0 μg/mL, and 0.02 μg/mL was used as the cut-off concentration.

**Text S2: Ethical oversight over the Population-based HIV Impact Assessment (PHIA) surveys in participating countries.**

The Eswatini Scientific and Ethics Committee, the National Health Science Research Committee Malawi, the National Health Research Ethics Committee Lesotho, the National Health Research Ethics Committee Lesotho, the Tropical Diseases Research Centre Ethics Review Committee, Zambia, the Medical Research Council of Zimbabwe, and the Institutional Review Boards at the Centers for Disease Control and Prevention (CDC; Atlanta, GA) and Columbia University Medical Center (New York, NY) approved the PHIA surveys.

**Table S1: Characteristics of HIV status disclosure among adults on antiretroviral therapy who participated in the Population-based HIV Impact Assessment (PHIA) survey in Lesotho, Malawi, Eswatini, Zambia and Zimbabwe (2015–2017).**

|  | **Lesotho** | **Malawi** | **Eswatini** | **Zambia** | **Zimbabwe** | **Total** |
| --- | --- | --- | --- | --- | --- | --- |
|  | N=2,177 | N=1,406 | N=2,025 | N=1,424 | N=2,168 | N=9,200 |
| Self-reported HIV status, n (%) | 2,177 (100.0) | 1,406 (100.0) | 2,025 (100.0) | 1,424 (100.0) | 2,168 (100.0) | 9,200 (100.0) |
| Positive | 2,065 (94.1) | 1,321 (93.6) | 1,967 (96.7) | 1,296 (90.7) | 2,051 (93.7) | 8,700 (93.1) |
| Negative | 61 (3.3) | 51 (3.6) | 45 (2.6) | 81 (5.7) | 81 (4.5) | 319 (4.3) |
| Never tested/unknown/refused | 51 (2.7) | 34 (2.9) | 13 (0.7) | 47 (3.6) | 36 (1.8) | 181 (2.5) |
| Disclosure to no one, n (%) | 2,065 (100.0) | 1,321 (100.0) | 1,967 (100.0) | 1,296 (100.0) | 2,051 (100.0) | 8,700 (100.0) |
| No | 1,997 (96.7) | 1,292 (97.6) | 1,910 (97.1) | 1,262 (97.7) | 2,002 (97.7) | 8,463 (97.5) |
| Yes | 68 (3.3) | 29 (2.4) | 57 (2.9) | 34 (2.3) | 49 (2.3) | 237 (2.5) |
| Disclosure to spouse/sex partner, n (%) | 2,065 (100.0) | 1,321 (100.0) | 1,967 (100.0) | 1,296 (100.0) | 2,051 (100.0) | 8,700 (100.0) |
| No | 1,007 (47.7) | 521 (40.2) | 1,045 (52.9) | 525 (39.8) | 824 (38.9) | 3,922 (41.2) |
| Yes | 1,058 (52.3) | 800 (59.8) | 922 (47.1) | 771 (60.2) | 1,227 (61.1) | 4,778 (58.8) |
| Disclosure to a doctor, n (%) | 2,065 (100.0) | 1,321 (100.0) | 1,967 (100.0) | 1,296 (100.0) | 2,051 (100.0) | 8,700 (100.0) |
| No | 1,759 (84.3) | 1,001 (75.7) | 1,898 (96.5) | 1,050 (80.0) | 1,664 (82.3) | 7,372 (81.1) |
| Yes | 306 (15.7) | 320 (24.3) | 69 (3.5) | 246 (20.0) | 387 (17.7) | 1,328 (18.9) |
| Disclosure to a friend, n (%) | 2,065 (100.0) | 1,321 (100.0) | 1,967 (100.0) | 1,296 (100.0) | 2,051 (100.0) | 8,700 (100.0) |
| No | 1,617 (77.7) | 1,095 (83.5) | 1,885 (95.6) | 1,098 (84.2) | 1,696 (84.0) | 7,391 (84.1) |
| Yes | 448 (22.3) | 226 (16.5) | 82 (4.4) | 198 (15.8) | 355 (16.0) | 1,309 (15.9) |
| Disclosure to a family member, n (%) | 2,065 (100.0) | 1,321 (100.0) | 1,967 (100.0) | 1,296 (100.0) | 2,051 (100.0) | 8,700 (100.0) |
| No | 540 (27.5) | 344 (25.8) | 660 (33.7) | 291 (22.6) | 507 (26.5) | 2,342 (26.0) |
| Yes | 1,525 (72.5) | 977 (74.2) | 1,307 (66.3) | 1,005 (77.4) | 1,544 (73.5) | 6,358 (74.0) |
| Abbreviations: N, Number of eligible participants; n, number of participants providing a valid response (weighted %). | | | | | | |

**Table S2: Prevalence of alcohol use disorder among adults on antiretroviral therapy who participated in the Population-based HIV Impact Assessment (PHIA) survey in Malawi, Eswatini, Zambia, and Zimbabwe (2015–2017).**

|  | **Total** |
| --- | --- |
|  | N=7,023 |
| AUDIT-C screening outcome, n (%) | 6,915 (100.0) |
| Negative | 6,418 (91.7) |
| Positive | 497 (8.3) |
| AUDIT-C score, n (%) | 6,915 (100.0) |
| 0 | 5,691 (80.1) |
| 1-2 | 573 (9.1) |
| 3-4 | 359 (5.6) |
| 5-8 | 248 (4.4) |
| 9-12 | 44 (0.8) |
| Based on Alcohol Use Disorder Identification scale (AUDIT-C)  Abbreviations: N, Number of eligible participants; n, number of participants providing a valid response (weighted %). | |
| In men, an AUDIT-C score of ≥4 and in women, a score of ≥3 is considered positive for hazardous drinking or active alcohol use disorder.  Lesotho was excluded from this analysis because AUDIT-C was not collected in this country. | |

**Table S3: Sensitivity analysis of rates of switching to second-line ART among HIV-positive adults who participated in the Population-based HIV Impact Assessment (PHIA) survey in Lesotho, Malawi, Eswatini, Zambia, and Zimbabwe (2015–2017).**

|  | **Assumed timing of switching** | | |
| --- | --- | --- | --- |
|  | 1.85 years after ART initiation | 6 months after ART initiation | Day of survey |
| **Country** | Annual rate in %  (95% CI) | Annual rate in %  (95% CI) | Annual rate in %  (95% CI) |
| Lesotho | 0.22 (0.14-0.36) | 0.22 (0.14-0.36) | 0.22 (0.14-0.36) |
| Malawi | 0.15 (0.08-0.35) | 0.15 (0.08-0.35) | 0.15 (0.08-0.35) |
| Eswatini | 0.72 (0.54-0.96) | 0.72 (0.55-0.97) | 0.69 (0.53-0.92) |
| Zambia | 0.73 (0.52-1.04) | 0.74 (0.53-1.05) | 0.70 (0.51-0.99) |
| Zimbabwe | 0.06 (0.02-0.33) | 0.06 (0.02-0.34) | 0.06 (0.02-0.33) |
| **Total** | 0.31 (0.25-0.40) | 0.31 (0.25-0.40) | 0.31 (0.24-0.39) |

Data are annual rates of switching to second-line antiretroviral therapy (ART) in percent and 95% confidence intervals (CI).
